# Sex-Specific Effects of a Maternal Obesogenic Diet High in Fat and Sugar on Offspring Adiposity, Growth, and Behavior

**DOI:** 10.3390/nu15214594

**Published:** 2023-10-29

**Authors:** Emily J. Mort, Sophie Heritage, Susan Jones, Abigail L. Fowden, Emily J. Camm

**Affiliations:** 1 Department of Physiology, Development and Neuroscience, University of Cambridge, Downing Street, Cambridge CB2 3EG, UK; 2 The Ritchie Centre, Hudson Institute of Medical Research, 27-31 Wright Street, Clayton, VIC 3168, Australia

**Keywords:** adiposity, high fat and sugar diet, dietary intake, obesity, behavioral neuroscience

## Abstract

With rising rates of human obesity, this study aimed to determine the relationship between maternal diet-induced obesity, offspring morphometrics, and behavior in mice. Pregnant and lactating female mice fed a diet high in fat and sugar (HFHS) commonly consumed by human populations showed decreased food, calorie, and protein intake but increased adiposity at the expense of lean mass. The pre-weaning body weight of the HFHS offspring was reduced for the first postnatal week but not thereafter, with HFHS female offspring having higher body weights by weaning due to continuing higher fractional growth rates. Post-weaning, there were minor differences in offspring food and protein intake. Maternal diet, however, affected fractional growth rate and total body fat content of male but not female HFHS offspring. The maternal diet did not affect the offspring’s locomotor activity or social behavior in either sex. Both the male and female HFHS offspring displayed reduced anxiety-related behaviors, with sex differences in particular aspects of the elevated plus maze task. In the novel object recognition task, performance was impaired in the male but not female HFHS offspring. Collectively, the findings demonstrate that maternal obesity alters the growth, adiposity, and behavior of male and female offspring, with sex-specific differences.

## 1. Introduction

Human epidemiological and experimental animal studies have shown that the adult phenotype can be programmed by sub-optimal intrauterine conditions resulting in intrauterine growth restriction such as under-nutrition, stress, and/or hypoxia [1,2]. More recently, evidence has grown that adverse intrauterine environments can also affect neurodevelopment, with consequences for adult behavior [3]. With increasing consumption of obesogenic diets globally, there has also been greater emphasis on over-nutrition and maternal obesity in developmental programming, as more women enter pregnancy with a high body mass index (BMI). In the UK, approximately 20–28% of women in antenatal care are classified as obese (BMI ≥ 30 kg/m^2^), while >30% are overweight [4], which has significant implications for a successful pregnancy outcome and long-term child health [5]. Compared to mothers with a healthy BMI (18.5–24.9 kg/m^2^), pregnant women with a high BMI are more prone to gestational diabetes mellitus and stillbirth, while their infants are at greater risk of abnormal birthweight and adult cardiometabolic dysfunction [5,6]. More recent epidemiological studies indicate that, by adolescence, these infants are also more likely to develop neurodevelopmental conditions such as intellectual disability, anxiety, autism spectrum disorder, attention deficit hyperactivity disorder, and schizophrenia [7,8]. However, whether these behavioral disorders are caused by maternal obesity during pregnancy and lactation and/or by a later obesogenic environment remains unclear.

Critical periods for programming offspring behavior can be studied more systematically in experimental animals like rodents, in which dietary intake, growth, and adiposity can be measured precisely throughout their lifespan. Maternal diet-induced obesity in rodents has been shown to influence locomotor activity, anxiety, and social-related behaviors and cognition in the offspring. However, these studies show conflicting outcomes [9,10,11,12]. They have also concentrated on high-fat diets rather than diets high in both fat and sugar, which are more commonly consumed by obese human populations [13]. Indeed, the high-fat diets used experimentally often have a lower sugar content than standard, control diets [14]. Detailed descriptions of maternal and offspring caloric and protein intake and of somatic growth and adiposity are not often reported in studies investigating the impact of a maternal obesogenic diet on behavioral outcomes of their adult offspring. In addition, many of these behavioral studies concentrate on the male offspring because of the potential variability in female data caused by estrus cycling [15].

With the greater emphasis now on investigating both sexes simultaneously [16], this murine study tested the hypothesis that maternal obesity induced by a diet high in fat and sugar has differential effects on the adult behavior of their male and female offspring fed a post-weaning control diet. Offspring behavior was assessed using the open field, elevated plus maze, social preference, and novel object recognition tasks with reference to the biometry, adiposity, and dietary intake of the offspring and their mothers.

## 2. Materials and Methods

### 2.1. Experimental Design

All animal experimentation was carried out under the UK Home Office Animals (Scientific Procedures) Act of 1986, following ethical approval by the University of Cambridge Animal Welfare and Ethical Review Body (AWERB, 4 June 2018). A total of 233 C57Bl/6J mice were studied. Of these, 106 were females purchased commercially for breeding (Charles River, Margate, UK), while the remainder were their offspring. All the mice were group-housed (*n* = 3–7 mice per cage) for most of the experimental period under a 12:12 h dark/light photocycle with ad libitum access to food and water. The majority of the dams and offspring (*n* = 178) were fed a standard rodent diet (RM3, Special Dietary Services [SDS], Witham, UK; 11% kcal fat; 62% kcal carbohydrates, of which 7% kcal is simple sugar; 27% kcal protein; and a water content of 10%). The remaining females used for breeding (*n* = 55) were fed a customized high-fat, high-sugar (HFHS) diet to induce obesity. This diet was made by combining high-fat diet pellets (D12451 diet, Research Diets Inc., Copenhagen, Denmark) with condensed milk (Carnation, Nestle, Gatwick, UK) and water to form patties that were baked at 55 °C for 46–48 h, as described previously [17]. The final nutritional composition of the HFHS diet was 38% kcal fat; 45% kcal carbohydrates, of which 33% kcal was simple sugars; and 17% kcal protein; and a water content of 12%. This diet was similar in composition to that consumed by obese women reported previously [18]. Both mouse diets were replenished every 48 h during the period of the study to ensure palatability.

Eight-week-old female mice were fed the standard or HFHS diet for 6 weeks before mating and throughout pregnancy and lactation (timeline, Supplementary Figure S1). Body weight was measured weekly before, during and after pregnancy on both diets. For a subset of the cages, intakes of food, kilocalories and protein were calculated by weighing food consumed in each cage over 24–48 h periods and totaling the food intake per week. Values are expressed as grams or kilocalories per day per mouse. After 6 weeks on the diet, a subset of animals in each dietary group were killed by cervical dislocation to measure body fat and lean mass by Dual Energy X-ray Absorptiometry (DEXA) scanning (Lunar PIXImus densitometer; GE Healthcare, Chicago, IL, USA) and the weights of individual fat deposits before pregnancy (*n* = 7 per group). The remaining females were mated with males fed the control diet. At day 18.5 days of pregnancy when the dams had been on their respective diets for 9–10 weeks (17–18 weeks of age), a further subset from each dietary group was killed by cervical dislocation to measure maternal fat deposit weights and DEXA body composition (term 20.5 days, *n* = 9 per group). The remaining control and HFHS-fed pregnant dams were then single-housed until their pups were weaned at postnatal (PN) day 21. Pups were sexed and weighed at PN2; ano-genital distance was measured with digital calipers for the determination of sex. All litters were then reduced to 6 pups per dam. Mean litter size at birth did not differ with maternal diet. Pups were then weighed again at PN7, PN14 and PN21 upon weaning. All post-partum dams were killed by cervical dislocation after weaning at approximately 20 weeks of age (after 12–13 weeks on their respective diets), with fat deposit weights and DEXA body composition measured in a subset of each dietary group.

At weaning, the offspring were group housed by sex and maternal diet, and fed the control diet. Thereafter, they were weighed weekly until behavioral assessments began post puberty at 13 weeks (PN91). Fractional growth rates (FGR) were calculated over specific time periods by dividing the weight increment over the period by the weight at the start of each period. In a subset of the offspring cages, intake of food, kilocalories and protein per mouse was measured weekly as grams or kilocalories per day. A total of 127 offspring were studied (*n* = 64 from control dams, *n* = 32 females, *n* = 32 males; *n* = 63 from HFHS dams, *n* = 32 females, *n* = 31 males). At the end of the behavioral assessments, all offspring were killed by cervical dislocation at 14 weeks (PN98) with DEXA body composition and/or fat deposit weights measured in a subset of both females and males in each dietary group.

### 2.2. Behavioral Testing

At intervals of 3–4 days, offspring carried out two different behavioral tasks. They were tested either in the elevated plus maze (EPM) followed by the novel object recognition (NOR) tasks, or with the combination of the open field (OF) and social preference (SP) tasks. Only one male and one female from a litter were tested in a particular combination of behavioral tasks. Female mice were tested in proestrus to account for the behavioral effects of estrus cycling [15,19]. Each testing session was recorded with a ceiling-mounted webcam and the videos analyzed manually blinded to the cohort where possible.

The OF and EPM tasks were used to assess novelty, locomotion, exploration, and anxiety and risk-taking behaviors [20]. In the OF arena, the number of grid lines crossed was used as an index of locomotion, while the time spent in the center and periphery and the number of rears were used as a measure of exploration and anxiety-related behavior [19]. For the EPM, the number of entries and time spent in the open and closed arms, the number of explorations to the end of the open arm, and the total number of rears were used as an index of exploration, anxiety, and risk-taking behaviors [19].

The SP task is designed to assess a mouse’s preference for interacting with a social stimulus versus a non-social stimulus [21]. The test mice were first habituated to the testing arena before being exposed to the unfamiliar sex- and strain-matched intruder mouse, as described previously [19]. The interaction times with the intruder (social stimulus -TS) and non-social stimulus (TNS) were quantified, and the social preference index (SPI) was calculated as follows:

SPI = (TS − TNS)/(TS + TNS)

The NOR task was used to evaluate cognition, particularly recognition memory [22]. During the acquisition phase, two identical objects were placed at diagonal corners of the test box. The retention phase of the test was set up identically to the acquisition phase, except that one of the familiar objects was replaced with a novel object. A discrimination index (DI) was calculated for each animal to indicate which object was favored as DI = [time spent investigating novel object—time spent investigating familiar object]/total time investigating objects. A preference for the novel or familiar object was indicated by a positive or negative DI, respectively.

### 2.3. Statistical Analysis

All statistical analyses were performed using GraphPad Prism (version 9.5.1 for Windows, GraphPad Software, San Diego, CA, USA). A Shapiro–Wilk normality test was used to examine if the data were normally distributed. The data are presented as mean ± standard deviation. A *t*-test or Mann–Whitney non-parametric test was used, as appropriate, to compare the biometric and behavioral measures between the two groups (control versus HFHS). A mixed effects analysis or two-way ANOVA followed by a Sidak’s post hoc test was used to compare the effects of diet and age on food intake and body mass. Statistical significance was accepted as *p* ≤ 0.05.

## 3. Results

### 3.1. Dietary Intake and Biometry of the Dams

**Figure 1:**
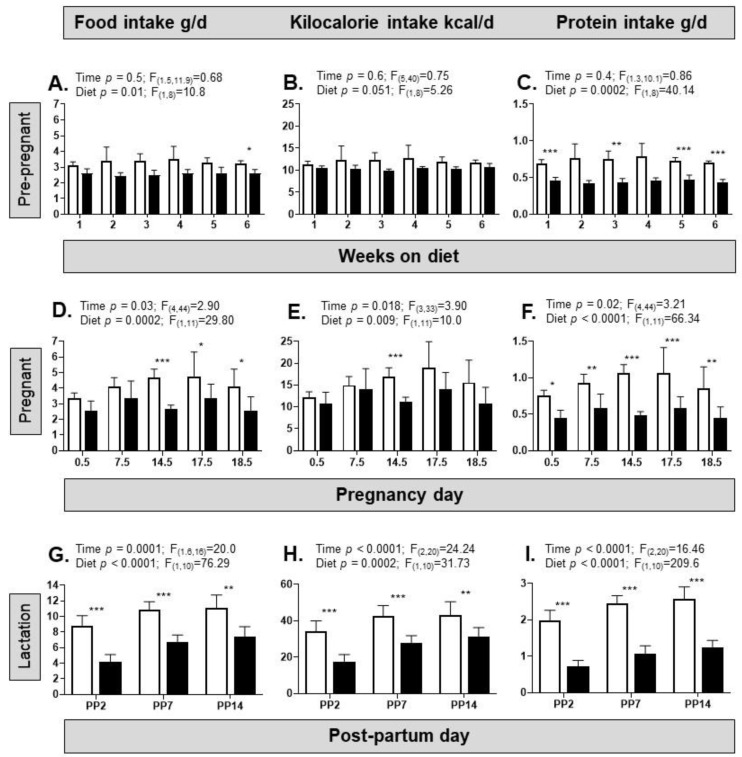
An obesogenic diet alters nutrient intake in pre-pregnant, pregnant, and lactating female mice. (**A**) Food intake (grams of food per day) in female mice fed either a control diet (from *n* = 5 cages, white columns) or a high-fat, high-sugar (HFHS, from *n* = 5 cages, black columns) diet for 6 weeks before pregnancy; (**B**) kilocalorie intake per day in the same groups of female mice (control, white columns; HFHS, black columns); (**C**) protein intake (grams of protein per day) in the same group of female mice (control, white columns; HFHS, black columns); (**D**–**F**) food intake, kcal intake, and protein intake in female mice fed either a control diet (from *n* = 7 cages, white columns) or a HFHS (from *n* = 6 cages, black columns) diet over each week while pregnant. Note that the *x*-axis scale is non-linear. (**G**–**I**) Food intake, kcal intake, and protein intake in female mice fed either a control diet (from *n* = 7 cages, white columns) or a HFHS (from *n* = 5 cages, black columns) diet on specific post-partum (PP) days during lactation. Note that the *x*-axis scale is non-linear. Two-way ANOVA results are given above each graph (interaction was not significantly different in all cases); post hoc test of diet effect indicated above bars, * *p* < 0.05, ** *p* < 0.01, *** *p* < 0.001.

The HFHS diet affected the nutrient intake of the dams pre-pregnancy and throughout pregnancy and lactation (Figure 1). Across the 6-week pre-pregnancy period, the HFHS diet significantly reduced the intake of food and protein, with a similar tendency for kilocalorie intake compared to controls (Figure 1A–C). Time per se did not affect any of the dietary intake of the dams pre-pregnancy (Figure 1A–C). During pregnancy, there were significant effects of both diet and pregnancy day on the intakes of food, calories, and protein (Figure 1D–F), with no interaction between diet and pregnancy day. Similarly, during lactation, both diet and day had significant effects on the intake of food, calories, and protein (Figure 1G–I), with no interaction between factors. Overall, these dietary intakes were lower in the HFHS diet condition, particularly later in pregnancy and throughout lactation (Figure 1D–I).

**Figure 2:**
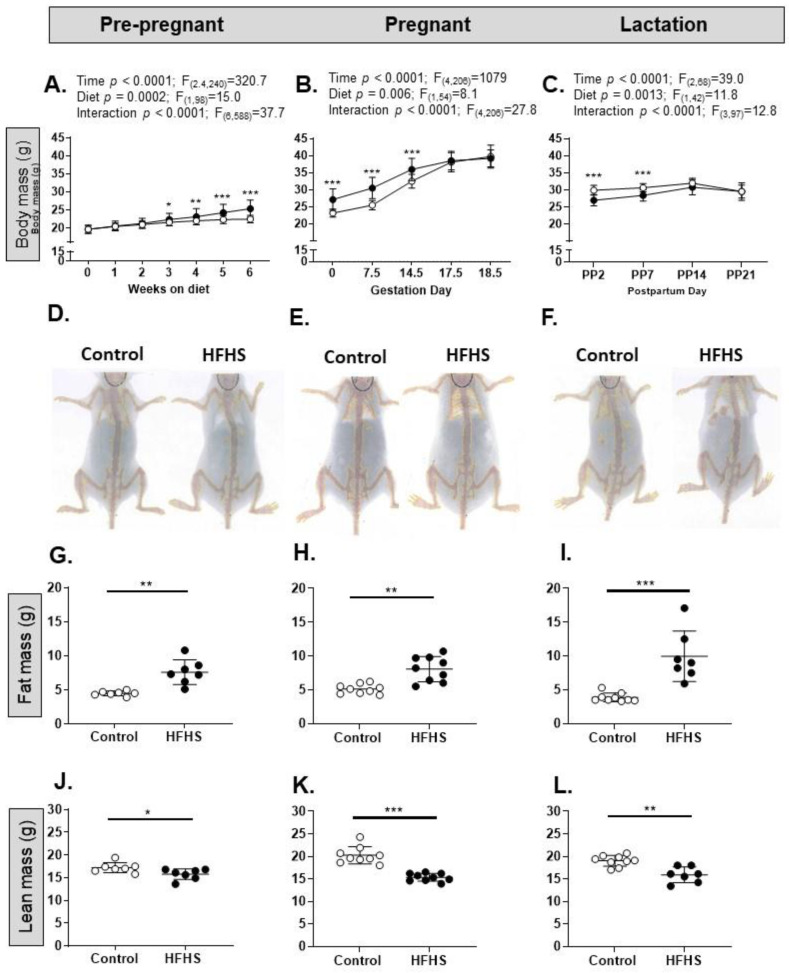
An obesogenic diet affects growth and adiposity in female mice. (**A**–**C**) Total body mass (g) of female mice on either a control (white symbols) or high-fat, high-sugar (HFHS, black symbols) diet, either pre-pregnant ((**A**); control *n* = 45, HFHS *n* = 55), pregnant ((**B**); control *n* = 35, HFHS *n* = 39), or lactating ((**C**); control *n* = 27, HFHS *n* = 17). Two-way ANOVA (**A**) or mixed effects analysis (**B**,**C**) results given above graphs; post hoc test of diet effect, * *p* < 0.05, ** *p* < 0.01, *** *p* < 0.001. (**D**) Example DEXA scans of female mice aged 14 weeks on the control diet (left) or HFHS diet (right); (**E**) example DEXA scans of female mice aged ~17 weeks on the control diet (left) or HFHS diet (right); (**F**) example DEXA scans of female mice aged ~20 weeks on the control diet (left) or HFHS diet (right); (**G**–**I**) total fat mass (g) in pre-pregnant ((**G**); *n* = 7; ** *p* = 0.0037, *t*-test), pregnant ((**H**); *n* = 9; ** *p* = 0.0012, *t*-test), and lactating ((**I**); *n* = 7–9; *** *p* = 0.0002, Mann–Whitney) female mice on a control (white symbols) or HFHS (black symbols) diet; (**J**–**L**) total lean mass (g) in pre-pregnant ((**J**); *n* = 7; * *p* = 0.035, *t*-test), pregnant ((**K**); *n* = 9; *** *p* < 0.0001, *t*-test), and lactating ((**L**); *n* = 7–9; ** *p* = 0.0023, *t*-test) female mice on a control or HFHS diet (control, white symbols; HFHS, black symbols).

The diet affected body weight, total body fat mass, and individual fat deposit masses in all three reproductive states (Figure 2; Table 1). Before pregnancy, there was a significant effect of both diet and time on total body mass, with a significant interaction between the two factors such that body weight increased more with age in the HFHS diet group (Figure 2A). Overall, these factors and their interactions remained significant during pregnancy and lactation (Figure 2B,C). The body weights of the HFHF dams remained significantly elevated until day 14.5 of pregnancy but were lower than the controls at post-partum (PP) day 2 and PP7 (Figure 2B,C), although body weight did not differ with diet by the end of pregnancy or lactation (Table 1). The total fat mass and weights of the individual fat deposits were significantly higher in the HFHS group than the control diet group at the expense of lean mass, irrespective of reproductive state (Figure 2G–L, Table 1). In addition, the adrenal glands were heavier in the HFHS group than in the control-fed pre-pregnant mice, but not during pregnancy or lactation, while the livers of the pregnant but not pre-pregnant or lactating dams were heavier in the HFHS diet group (Table 1).

### 3.2. Dietary Intake and Biometry of the Offspring

**Figure 3:**
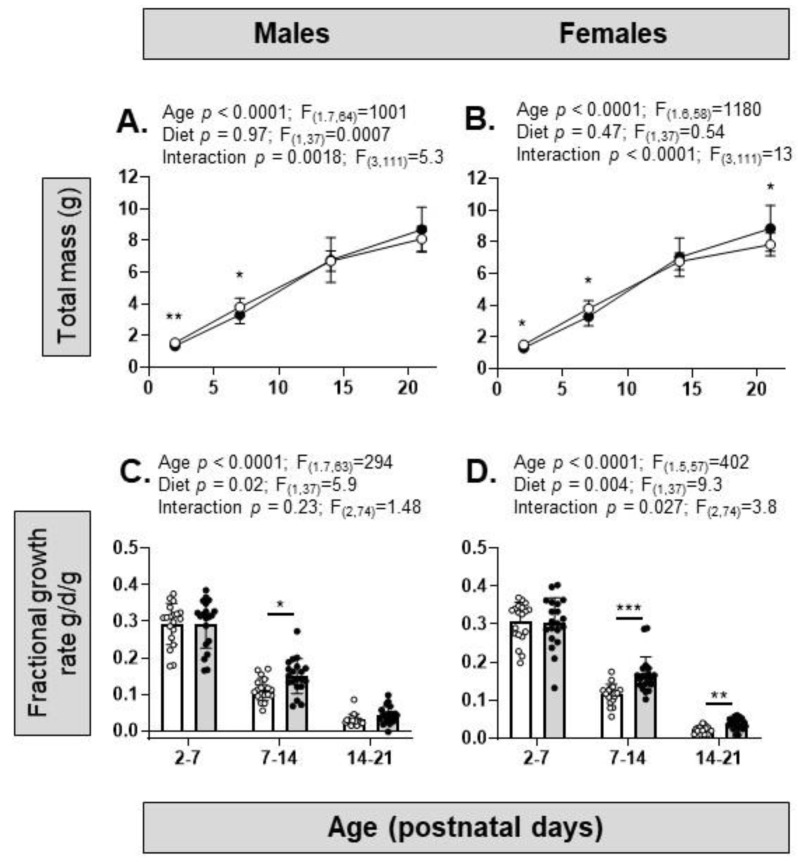
Pre-weaning growth in the offspring of dams on control or high-fat, high-sugar (HFHS) diets. (**A**) Total mass during postnatal development (postnatal day 2 to 21) in male offspring of mothers on a control (white symbols, *n* = 19) or HFHS (black symbols, *n* = 20) diet; (**B**) female offspring from mothers on a control (white symbols, *n* = 19) or HFHS (black symbols, *n* = 20) diet. Two-way ANOVA results given above graphs; post hoc test of diet effect, * *p* < 0.05, ** *p* < 0.01; (**C**) fractional growth rate in male offspring of mothers on a control (white symbols and bars, *n* = 19) or HFHS (black symbols and bars, *n* = 20) diet; (**D**) fractional growth rate in female offspring (control, white symbols and bars, *n* = 19; HFHS, black symbols and grey bars, *n* = 20). Two-way ANOVA results given above graphs; post hoc test of diet effects, * *p* < 0.05, ** *p* < 0.01, *** *p* < 0.001.

Overall, during pre-weaning development, there was a significant effect of age but not diet on body weight (Figure 3A,B). There was a significant interaction between age and diet, with significantly lower body weights in the HFHS pups than the control pups of both sexes at PN2 and PN7, as well as higher body weights in the females but not the males at PN21 (Figure 3A,B). The FGR of the pre-weaning pups decreased significantly with age and was higher overall in the HFHS pups than control pups of both sexes, particularly after PN7, remaining higher for longer in the females (Figure 3C,D).

**Figure 4:**
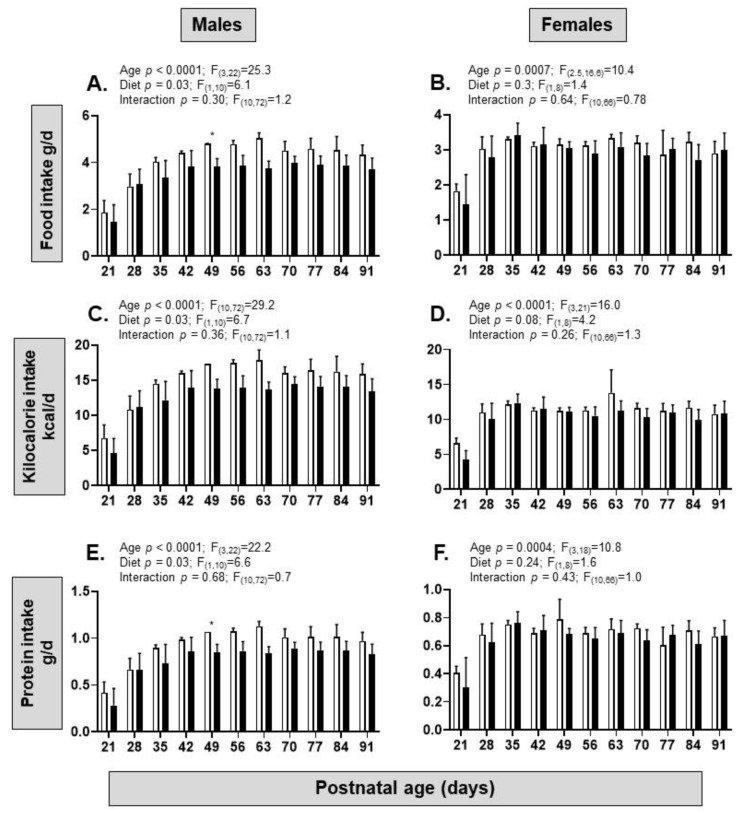
Post-weaning food intake of offspring from postnatal day 21 to 91. (**A**) Food intake (grams of food per day) in male mice from mothers on a control (from *n* = 2–5 cages, white columns) or high-fat, high-sugar (HFHS, from *n* = 6–7 cages, black columns) diet measured between postnatal days 21 to 91; (**B**) food intake (grams of food per day) in female mice from mothers on a control (from *n* = 3–5 cages, white columns) or HFHS diet (from *n* = 5 cages, black columns) measured between postnatal days 21 to 91; (**C**,**D**) kilocalorie intake (kcal per day) in the same groups of male and female mice from mothers on a control (white symbols) or HFHS diet (black symbols); (**E**,**F**) protein intake (grams of protein per day) in in the same groups of male and female mice from mothers on a control (white symbols) or HFHS diet (black). Mixed effects analysis results shown above graphs; post hoc test of diet effect at specific ages, * *p* < 0.05.

After weaning, food intake, kilocalories, and protein increased with age from PN21 to PN91 in both sexes but were only affected by maternal diet in the males with lower intakes in the HFHS group than the control offspring. There were no interactions between age and diet on the dietary intake in either sex (Figure 4A–F).

**Figure 5:**
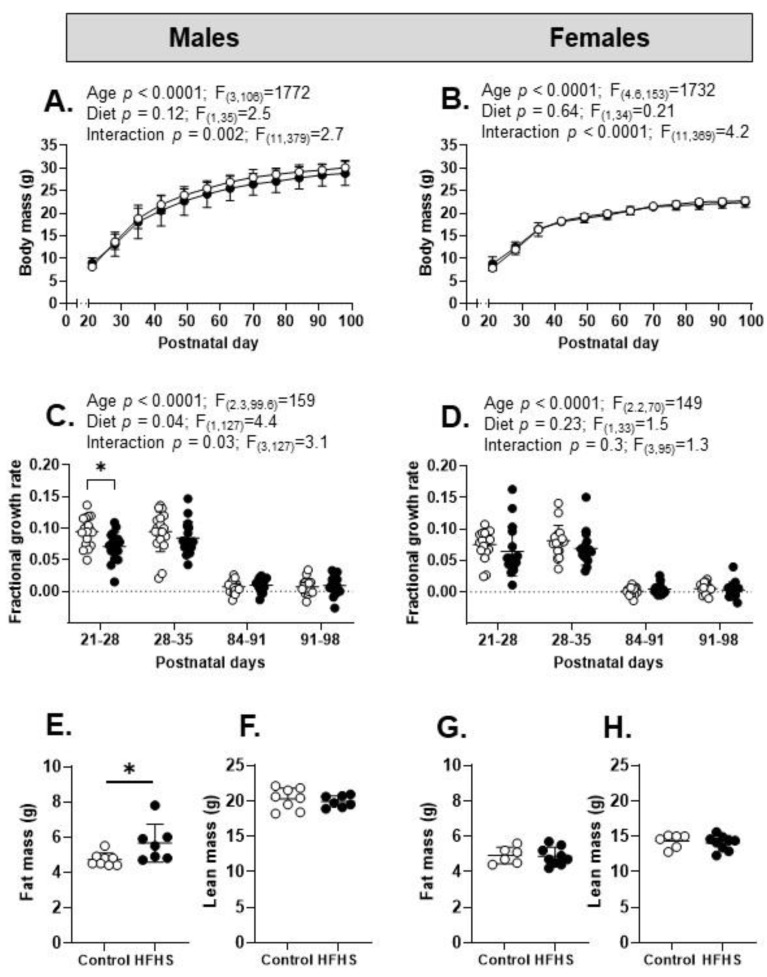
Post-weaning growth and fat deposition in the offspring of mothers on control or high-fat, high-sugar (HFHS) diets. (**A**,**B**) Body mass (g) in male and female offspring from mothers on a control (*n* = 18–19, white symbols) or HFHS (*n* = 18, black symbols) diet. Mixed effects analysis (**A**,**B**) results are given above graph; post hoc tests show no diet effects at specific ages. (**C**,**D**) Fractional growth rate with respect to weekly postnatal periods in male and female offspring from mothers on a control (*n* = 18, white symbols) or HFHS (*n* = 17, black symbols) diet. Mixed effects analysis (**C**,**D**) results given above graph; post hoc tests in males (P21–28: * *p* < 0.01); no effect of diet in females. (**E**,**F**) Total fat mass and lean mass (g) in male offspring from mothers on a control (*n* = 8) or HFHS (*n* = 7) diet (symbols as before); (**G**,**H**) fat mass and lean mass (g) in female offspring from mothers on a control (*n* = 6) or HFHS (*n* = 9) diet (* *p* < 0.05, unpaired *t*-test; symbols as before).

There was a significant effect of increasing offspring age on post-weaning body weight and a significant interaction with maternal diet overall in both sexes (Figure 5A,B), although body weight did not differ with maternal diet in either sex at PN98 (Table 1). Post-weaning FGR decreased with age in both sexes and was also lower in male but not female HFHS offspring during the first week after weaning (Figure 5C,D). At PN98, total fat mass was significantly higher in the male but not female HFHS offspring, with no differences in lean mass weight with maternal diet in either sex (Figure 5E–H). The weights of the gonadal, retroperitoneal, and perirenal fat deposits were all significantly greater in the HFHS than the control male offspring, whereas in the HFHS females, only the perirenal fat weighed more than the control values (Table 1) The adrenal weight was lower in the HFHS than the control offspring in males but not females (Table 1). At PN98, maternal diet had no effect on the weights of the brain, liver, or heart of the offspring of either sex (Table 1).

### 3.3. Behavior and Cognition Function of the Adult Offspring

**Figure 6:**
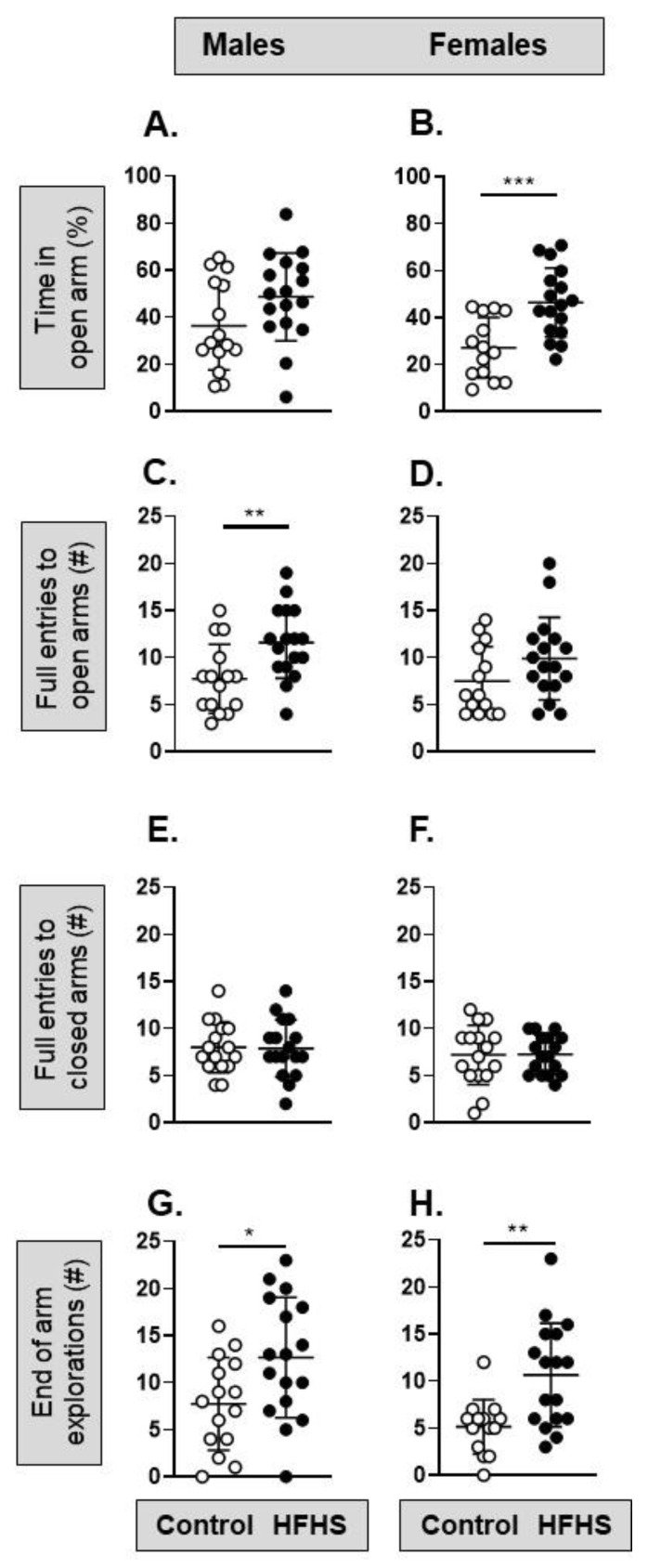
Pre-weaning effect of a maternal obesogenic diet on adult offspring behavior in the elevated plus maze (EPM). (**A**) The percentage of time spent in the open arm of the EPM by male mice from mothers on a control (*n* = 16, white symbols) or high-fat, high-sugar (HFHS; *n* = 17, black symbols) diet (*p* = 0.073, *t*-test); (**B**) percentage time spent in the open arm for female mice from mothers on a control (*n* = 14) or HFHS diet (*n* = 17; *** *p* = 0.0006, *t*-test; symbols as for male mice); (**C**) number of full entries into the open arm made by male mice from mothers on a control (*n* = 15) or HFHS diet (*n* = 17; ** *p* = 0.0065, *t*-test); (**D**) full entries into the open arm made by female mice (*p* = 0.15, Mann–Whitney); (**E**,**F**) number of full entries into the closed arm made by male (*p* = 0.91, *t*-test) and female (*p* = 0.96, *t*-test) mice from mothers on a control or HFHS diet; (**G**,**H**) number of explorations to the end of the open arm made by male (* *p* = 0.022, *t*-test) and female (** *p* = 0.0015, *t*-test) mice from mothers on a control or HFHS diet. #, number.

In the OF test, there were no significant differences in offspring locomotion (number of lines crossed), anxiety-related behavior (entries into the center or as duration in the center), or exploratory rearing with maternal diet in either sex (Supplementary Figure S2). In the EPM task, the female but not male offspring of the HFHS dams spent significantly more time in the open arm (Figure 6A,B), while the male but not female offspring of the HFHS dams made significantly more full entries into the open arm (Figure 6C,D) than the offspring of the control dams. In both sexes, the HFHS offspring made more explorations to the end of the open arm than their control counterparts (Figure 6G,H).

**Figure 7:**
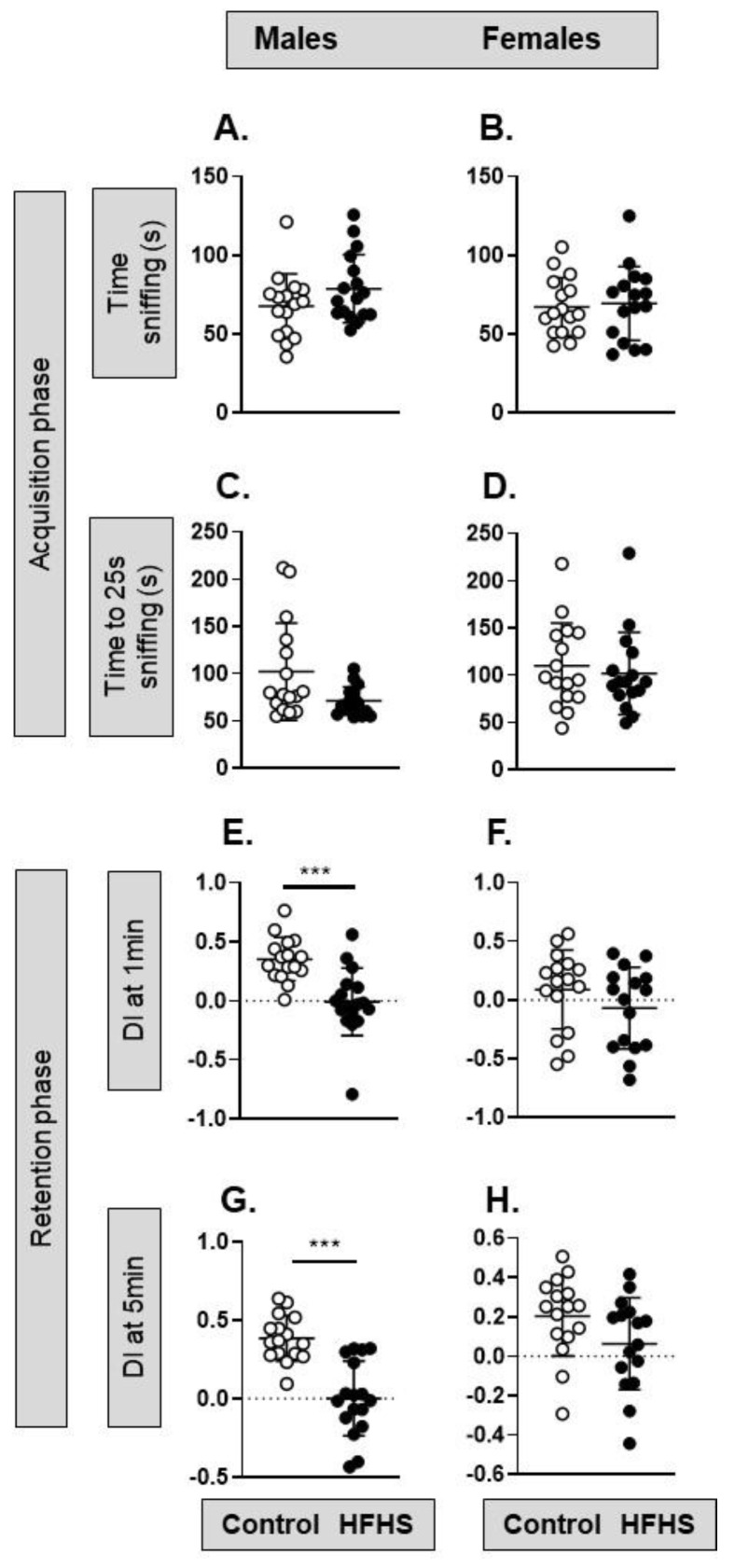
Effects of a maternal obesogenic diet on adult offspring behavior in the novel object recognition task. (**A**–**D**) Acquisition phase: (**A**) the time spent sniffing two identical objects by male mice from mothers on a control (*n* = 16, white symbols) or high-fat, high-sugar (HFHS; *n* = 17, black symbols) diet (*p* = 0.136, *t*-test); (**B**) the time sniffing two identical objects spent by female mice from mothers on a control (*n* = 16) or high-fat, high-sugar (HFHS; *n* = 16) diet (*p* = 0.78, *t*-test; symbols as for male mice); (**C**) time taken to reach 25 s of sniffing the two identical objects by male mice from mothers on a control (*n* = 16) or HFHS diet (*n* = 17; *p* = 0.076, *t*-test); (**D**) time taken to reach 25 s of sniffing the two identical objects by female mice from mothers on a control (*n* = 16) or HFHS diet (*n* = 16; *p* = 0.356, Mann–Whitney). (**E**–**H**) Retention phase: (**E**,**F**) discrimination index score of male (*** *p* = 0.0002, *t*-test; *n* = 16–17) and female (*p* = 0.47, *t*-test; *n* = 12–16) mice from mothers on a control or HFHS diet in the first minute of object exploration; (**G**,**H**) discrimination index score of male (*** *p* < 0.0001, *t*-test; *n* = 16–17) and female (*p* = 0.104, *t*-test; *n* = 12–16) mice from mothers on a control or HFHS diet after 5 min of object exploration.

Maternal diet has no significant effect on the social preference of either sex (Supplementary Figure S3). In the NOR task, there was no significant difference in the time spent sniffing two identical objects during the acquisition phase in either the male or female offspring of the HFHS dams (Figure 7A–D). In the retention phase of the NOR task, DI differed significantly with maternal diet in the male but not female offspring (Figure 7E–H). The control males and females had a positive DI due to spending more time sniffing the novel object, while the male HFHS offspring had a DI close to zero, indicating equal time spent sniffing the familiar and novel object (Figure 7E,F). Maternal diet had no effect on the retention phase of the NOR task in the female offspring (Figure 7F,H).

## 4. Discussion

This study demonstrates that feeding female mice a HFHS diet reduces food intake and increases adiposity both before and throughout pregnancy and lactation. This maternal HFHS diet also results in sex-specific effects on the growth trajectory, adiposity, anxiety-related behavior, and cognitive function of their adult offspring, which are summarized in Table 2.

### 4.1. Dam Food Intake and Biometry

Females fed the HFHS diet consumed significantly less food, which resulted in reduced calorie and protein intake throughout pregnancy and lactation. Despite the decreased dietary intake, the females fed the HFHS diet significantly increased their adiposity in all three reproductive states, which was associated with an increased body mass until near the end of pregnancy. The body mass of the HFHS dams fell during the early stages of lactation but increased to control values by the end of lactation when the body fat content was still 100% greater in the HFHS than the control dams. Previous studies using similar HFHS diets have also shown increased body weight and adiposity during pregnancy and lactation, with some of the greatest increases in fat mass seen in dams that had ad libitum access to the highly calorific sweetened condensed milk [23,24]. In the current study, increased fat accumulation was accompanied by a loss of lean mass, irrespective of reproductive state, which may reflect, in part, the reduced dietary and protein intake. With less lean mass and more fat, the HFHS mice may have had a reduced basal metabolic rate and expenditure of energy for processes such as locomotion and thermoregulation, which would contribute to a lower calorie requirement [25,26]. Indeed, the increased insulation provided by the enhanced fat deposition may have reduced the energy requirement to maintain core temperature. In addition, the enhanced fat deposition in the HFHS dams may have increased concentrations of leptin, the anorexigenic peptide, consistent with their reduced food intake. Indeed, increased leptin concentrations have been observed in previous studies of pregnant HFHS dams depending on their stage of pregnancy [17,27].

### 4.2. Offspring Food Intake and Biometry

In human populations, obesity during pregnancy can affect intrauterine growth, resulting in infants who are either small for their gestational age, growth restricted, or large for their gestational age [28]. In the current study, the body mass of both the female and male HFHS pups was reduced during the first week of life, which may reflect the reduced maternal kilocalorie and protein intake during pregnancy and/or in early lactation, as well as the transient fall in postpartum maternal weight with the onset of lactation. Previous studies using maternal low-protein diets and the current HFHS diet have shown fetal growth restriction at the end of gestation in association with impaired placental growth and morphology [17,29]. During the suckling period, HFHS pups exhibited catch-up growth irrespective of sex, a phenomenon commonly seen in growth-restricted neonates in utero [30]. The accelerated growth during suckling was maintained for longer in the HFHS females and stopped at weaning in both sexes, when the offspring were transferred to the control diet. Compared to the controls, the male but not female HFHS offspring reduced their fractional growth rate in the first week post-weaning and consumed less food, kilocalories, and protein throughout the 14-week postnatal study period without a significant decrease in their body mass. Despite this, the adult male HFHS offspring had a higher fat mass than the controls, while the fat mass of the adult HFHS females was unaffected by maternal diet. These differences in offspring growth trajectory and adult body composition suggest that male HFHS offspring, in particular, are probably programmed to be more metabolically efficient and/or more sedentary in their home environment. Further studies are therefore required to understand the determinants of energy balance in the adult HFHS offspring.

A number of previous human epidemiological and animal experimental studies have shown increased offspring fat deposition in response to maternal obesity during pregnancy, but this is most commonly associated with offspring hyperphagia and an increased body mass from weaning [23,31]. In many of the experimental studies in rodents, maternal obesity was induced by feeding high-fat (HF) rather than HFHS diets, with the offspring often studied at older ages than in the current study. Young adult offspring of obese mothers have been shown to increase their fat deposition in the absence of increased body weight in rats and sheep [32,33], in line with the current findings. The outcome of maternal obesity on offspring morphometry, therefore, appears to depend on a range of factors including species, offspring sex and age, and the specific maternal and post-weaning diet. The current findings suggest that offspring appetite, metabolism, and/or growth regulation can be programmed in a sex-linked manner by early life exposure to maternal diet-induced obesity.

### 4.3. Offspring Behavior and Cognitive Function

The current study clearly shows that aspects of offspring behavior and cognitive function are altered in both sexes by a maternal HFHS diet and concomitant obesity. Multiple measures obtained from the EPM task indicated reduced anxiety-like behavior in both male and female HFHS offspring, as evidenced by increased entries into the anxiogenic open arm environment and a greater number of visits to the distal end of the open arm. Previously, increased [34,35], decreased [36,37], or no change [38] in anxiety-related behaviors have been reported for the offspring of obese dams, which may relate to offspring age [36,37]. The greater frequency with which the HFHS offspring travelled to the distal ends of the open arm of the EPM in the current study could also be interpreted as increased risk-taking behavior. In a human study, an association between being overweight/obese and engaging in risky behaviors has been reported in adolescents [39] and warrants further investigation in the HFHS mouse offspring.

Male but not female HFHS offspring displayed a deficit in object recognition memory compared to control offspring. This finding aligns with the available literature on compromised recognition memory in the adult offspring of mothers with obesity [40,41,42,43]. In contrast, there was no effect of the maternal HFHS diet on offspring social behavior. Recent studies support the current finding [36,44], while others have reported maternal HF diet-induced disruptions to social processes in the offspring [45]. The observed effects of maternal diet-induced obesity on offspring social behavior may be heavily dependent on the age and sex of the offspring tested [44]. An interplay between social and anxiety-related behavior may explain why the HFHS offspring were comfortable interacting with the intruder mouse, with lower anxiety-related behavior in the HFHS offspring facilitating approach to and interaction with an unknown intruder mouse. Further behavioral analysis is needed to understand the contribution of anxiety in social situations for HFHS offspring.

In the current study, a maternal HFHS diet from pre-pregnancy through to lactation did not affect offspring locomotor activity in the open field arena. This finding agrees with several previous studies of maternal HF models [42,46]. On the other hand, a maternal HF diet has been shown to lead to hyperactivity in male offspring, along with increased speed of movement within the open field arena [36,41]. However, data from juvenile and adult offspring in experimental studies of maternal obesity suggest that enhanced locomotor activity is only temporary and diminishes with age [10], in agreement with the current findings in the adult HFHS offspring.

Determining the primary mechanism of developmental programming of adult behavior by the maternal HFHS diet is difficult due to the complexity of disentangling the maternal obesogenic milieu from the intrauterine and subsequent postnatal environments. Indeed, the current HFHS diet is known to cause sex-specific differences in the feto–placental phenotype, with alterations in the fetal nutrient supply and growth [17] which may contribute to the disparities observed postnatally between the male and female offspring reported here. In the male HFHS offspring, there were changes in the post-weaning dietary intake, growth profile, and adult adiposity, while in the female HFHS offspring, only the neonatal growth trajectory was altered relative to the controls. The changes in anxiety-related behaviors in both sexes of the HFHS offspring may, therefore, indicate a dependence on the combined effects of maternal obesity and the postnatal growth profile, independent of offspring adiposity or sex hormone environment, while the memory deficit of the male offspring may depend more heavily on their increased adiposity and testosterone production. Multiple interrelated mechanisms have been proposed to underlie offspring neurodevelopmental programming by diet-induced maternal obesity, including oxidative stress and inflammation, altered hypothalamic–pituitary–adrenal axis activity, dysregulation of dopaminergic and serotonergic signaling and reward circuitry, perturbations in growth factor-mediated synaptic plasticity, and impaired insulin, glucose, and leptin signaling in the brain (see [7]). For instance, the reduction in adrenal mass in the male HFHS offspring may affect corticosterone production and/or hypothalamic–pituitary–adrenal axis activity, resulting in blunted stress responsiveness and lowered anxiety. Increased leptin availability due to the increased adiposity of HFHS male offspring may also influence their phenotype [47]. Recent evidence supports the role of leptin in higher cognitive functions, particularly hippocampal-dependent learning and memory [48].

## 5. Conclusions

Collectively, the current data show that a HFHS diet fed before and throughout mouse pregnancy and lactation alters maternal dietary intake and body composition, with sex-specific consequences for offspring somatic growth, adiposity, anxiety-related behavior, and spatial memory. Maternal diet-induced obesity, therefore, has widespread programming effects on offspring development, with important implications for the long-term impacts of current obesogenic diets on the phenotypical diversity of human populations.

## Figures and Tables

**Table 1:** Post-mortem organ weights and fat deposition in pre-pregnant, pregnant, and lactating female mice, and of their female and male offspring fed a control diet post weaning. *** *p* < 0.01, ** *p* < 0.02, * *p* < 0.05, *t*-test or Mann–Whitney test. NA = not available.

	Dams						Offspring			
	Pre-Pregnant		Pregnant		Lactation		Females		Males	
	Control (*n* = 7)	HFHS (*n* = 7)	Control (*n* = 9)	HFHS (*n* = 9)	Control (*n* = 14–26)	HFHS (*n* = 10–18)	Control (*n* = 9–16)	HFHS (*n* = 13–16)	Control (*n* = 11–16)	HFHS (*n* = 13)
Total body weight (g)	22.4 ± 0.5	25.3 ± 0.8 ***	41.4 ± 3.5	39.3 ± 1.8	29.5 ± 2.0	29.8 ± 3.2	22.8 ± 0.6	22.3 ± 1.1	29.9 ± 1.5	28.8 ± 2.8
Brain (mg)	460 ± 18	460 ± 10	463 ± 24	442 ± 36	454 ± 29	454 ± 19	447 ± 9	453 ± 17	453 ± 16	442 ± 26
Liver (g)	1.20 ± 0.10	1.10 ± 0.10	1.9 ± 0.21	2.30 ± 0.30 **	2.12 ± 0.25	2.00 ± 0.32	1.11 ± 0.12	1.04 ± 0.13	1.49 ± 0.23	1.36 ± 0.23
Heart (mg)	120 ± 18	110 ± 23	152 ± 36	146 ± 12	211 ± 43	206 ± 102	121 ± 14	127 ± 11	168 ± 19	173 ± 31
Adrenal (mg)	7.8 ± 1.3	9.7 ± 1.3 *	6.8 ± 1.8	9.0 ± 5.0	7.7 ± 1.9	7.4 ± 2.3	6.3 ± 1.2	6.0 ± 1.0	5.0 ± 1.0	3.8 ± 0.6 ***
Gonadal fat (mg)	NA	NA	294 ± 76	484 ± 212 **	341 ± 90	1100 ± 448 ***	501 ± 102	514 ± 166	431 ± 104	579 ± 199 *
Retroperitoneal fat (mg)	37 ± 8	120 ± 63 ***	67 ± 15	156 ± 61 ***	55 ± 19	208 ± 105 ***	76 ± 15	72 ± 24	82 ± 29	150 ± 97 ***
Perirenal fat (mg)	46 ±13	110 ± 70 ***	135 ± 54	216 ± 71 **	132 ± 31	300 ± 162 ***	109 ± 35	143 ± 31 **	49 ± 19	80 ± 24 ***

**Table 2:** Summary of the effects of a maternal HFHS diet on dietary intake, body mass and composition, and on behavior and cognitive function of male and female offspring. FGR, fractional growth rate; PN, postnatal day. ↓, reduction; ↑ increase; ↔ no change.

	Male Offspring	Female Offspring
Dietary intake	↓ food intake ↓ calorie intake ↓ protein intake	↔ food intake ↔ calorie intake ↔ protein intake
Body mass and growth rate	↓ PN2–7 ↑ FGR PN7–14 ↓ FGR PN21–28	↓ PN2–7 ↑ FGR PN7–21 ↔ FGR PN21–28
Body composition	↑ fat mass ↔ lean mass ↑ gonadal mass ↑ retroperitoneal mass ↑ perirenal fat mass	↔ fat mass ↔ lean mass ↔ gonadal mass ↔ retroperitoneal mass ↑ perirenal fat mass
Locomotor activity	↔	↔
Anxiety-related behaviors	↑ open arm exploration ↔ % time in open arm ↑ full entries into open arm	↑ open arm exploration ↑ % time in open arm ↔ full entries into open arm
Social behavior	↔	↔
Cognition	↓ object recognition memory	↔

## Data Availability

The data presented in this study are openly available in Apollo at https://doi.org/10.17863/CAM.102134.

## Supplementary Materials

The following supporting information can be downloaded at: https://www.mdpi.com/article/10.3390/nu15214594/s1, Figure S1: Representation of the experimental timeline; Figure S2: Effect of a maternal obesogenic diet on adult offspring behavior in the open field; Figure S3: Effect of a maternal obesogenic diet on adult offspring social interaction behavior.
