# Supplementary material for: Sex-Specific Effects of a Maternal Obesogenic Diet High in Fat and Sugar on Offspring Adiposity, Growth, and Behavior
Source: Nutrients. 2023 Oct 29;15(21):4594. doi: 10.3390/nu15214594 (PMC10648016; doi:10.3390/nu15214594)
Supplement: Supplementary file 1 [file nutrients-15-04594-s001.zip › Mort Nutrients Revision 1 Supplementary Figure1.pdf]

Supplementary Figure 1

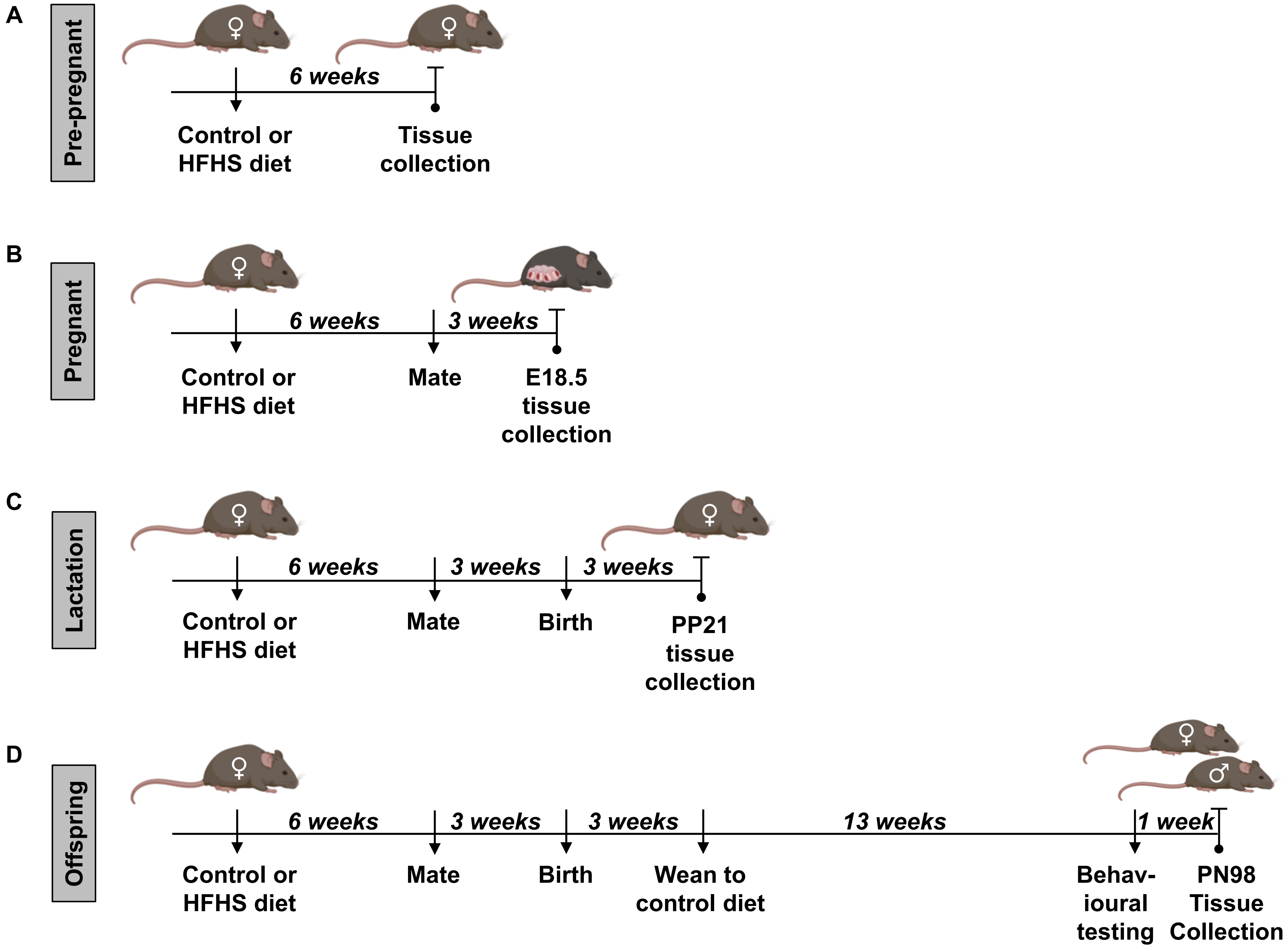

Supplementary Figure 1. Representation of the experimental timeline, showing the timing of tissue collection in pre-pregnant mice (A), pregnant mice (B), at lactation (C), and in adult offspring (D). E, embryonic day; PN, postnatal day; PP, post-partum day.
