# Supplementary material for: Sex-Specific Effects of a Maternal Obesogenic Diet High in Fat and Sugar on Offspring Adiposity, Growth, and Behavior
Source: Nutrients. 2023 Oct 29;15(21):4594. doi: 10.3390/nu15214594 (PMC10648016; doi:10.3390/nu15214594)
Supplement: Supplementary file 1 [file nutrients-15-04594-s001.zip › Mort Nutrients Revison 1 Supplementary Figure2.pdf]

Supplementary Figure 2

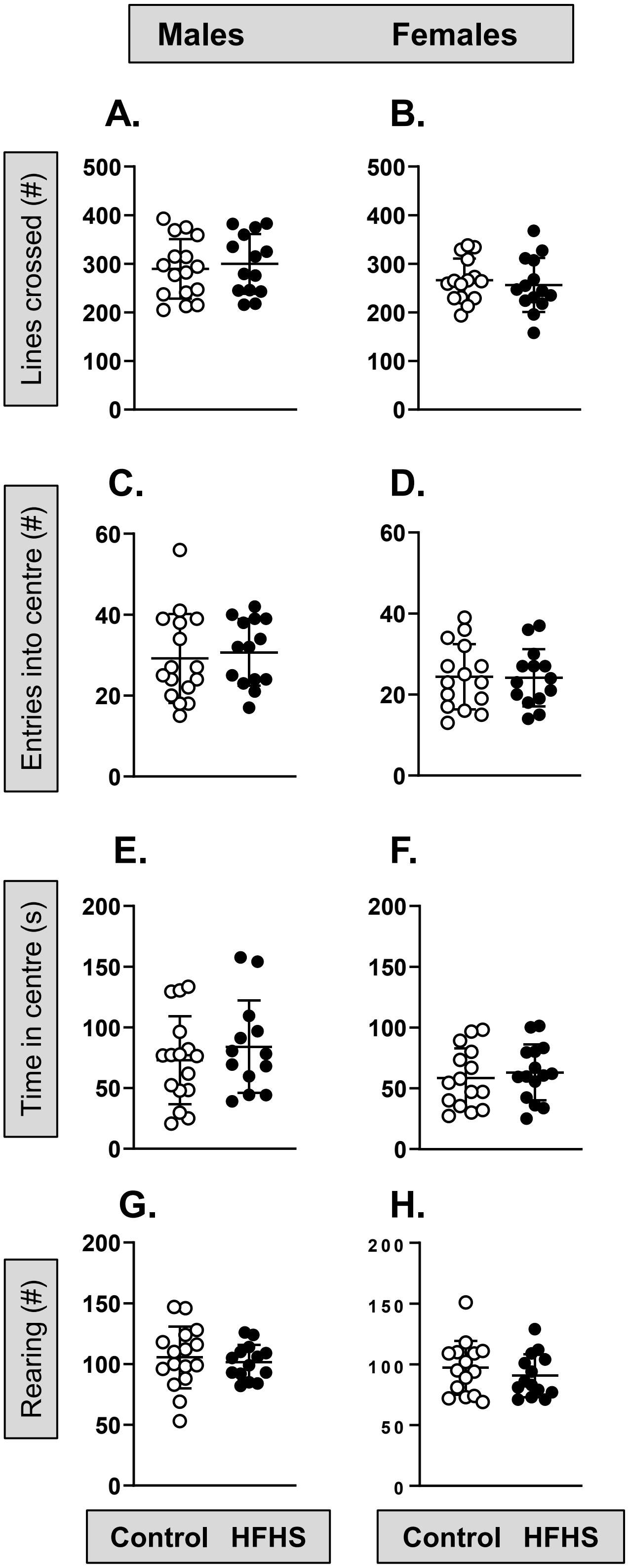

Supplementary Figure 2. Effect of a maternal obesogenic diet on adult offspring behaviour in the open field. (A) The number of lines crossed in the open field by male mice on a control (n=16, white symbols) or high fat, high sugar (HFHS; n=14, black symbols) diet (p=0.65, t-test); (B) Lines crossed by female mice on a control (n=15, white symbols) or HFHS (n=14) diet (p=0.61, black symbols, t-test); (C, D) Entries into the centre of the open field by male (p=0.67, t-test) and female (p=0.93, t-test) mice on a control (white symbols) versus a HFHS diet (black symbols); (E, F) Time (seconds) spent in the centre of the open field by (p=0.43, t-test) and female (p=0.59, t-test) mice on a control (white symbols) or HFHS (black symbols) diet. G, H. Number of rears made in the open field by male (p=0.61, t-test) and female (p=0.40, t-test) mice on a control (white symbols) or HFHS (black symbols) diet.
