# Supplementary material for: Sex-Specific Effects of a Maternal Obesogenic Diet High in Fat and Sugar on Offspring Adiposity, Growth, and Behavior
Source: Nutrients. 2023 Oct 29;15(21):4594. doi: 10.3390/nu15214594 (PMC10648016; doi:10.3390/nu15214594)
Supplement: Supplementary file 1 [file nutrients-15-04594-s001.zip › Mort Nutrients Revsion 1 Supplementary Figure3.pdf]

Supplementary Figure 3

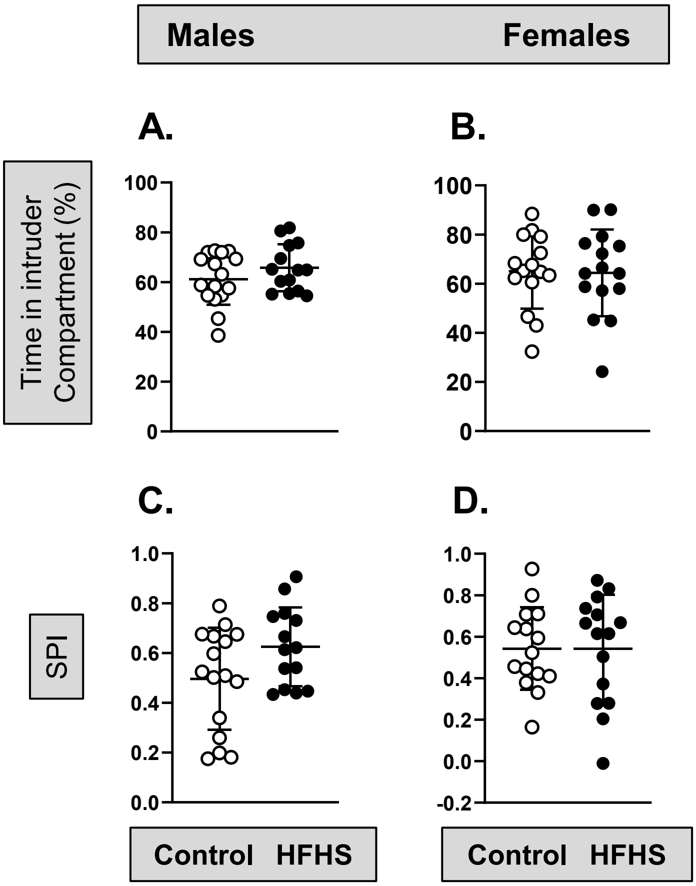

Supplementary Figure 3. Effect of a maternal obesogenic diet on adult offspring social interaction behaviour. (A) Percentage time spent in the intruder mouse compartment by male mice from mothers on the control (n=16, white symbols) or high fat, high sugar (HFHS; n=14, black symbols) diet (p=0.22, t-test); (B) Percentage time spent in the intruder mouse compartment by female mice from mothers on the control (n=15, white symbols) or HFHS (n=15, black symbols) diet (p=0.91, t-test); (C, D) The social preference index, a measure of the time spent interacting with the intruder mouse versus the object, in male mice (p=0.063, t-test) and female mice (p=0.99, t-test) from mothers on the control (white symbols) or HFHS (black symbols) diet.
